# Fluorine-containing substituents: metabolism of the α,α-difluoroethyl thioether motif

**DOI:** 10.3762/bjoc.15.144

**Published:** 2019-06-28

**Authors:** Andrea Rodil, Alexandra M Z Slawin, Nawaf Al-Maharik, Ren Tomita, David O’Hagan

**Affiliations:** 1 School of Chemistry, University of St Andrews, North Haugh, St Andrews, KY16 9ST, UK; 2 An Najah National University, Nablus, Palestine

**Keywords:** *Cunninghamella elegans*, cytochrome P450, fluorinated substituents, organofluorine metabolism, sulfoxidation

## Abstract

We report the metabolism of the recently introduced α,α-difluoroethyl thioether motif to explore further its potential as a substituent for bioactives discovery chemistry. Incubation of two aryl–SCF_2_CH_3_ ethers with the model yeast organism *Cunninghamella elegans,* indicates that the sulfur of the thioether is rapidly converted to the corresponding sulfoxide, and then significantly more slowly to the sulfone. When the substrate was (*p-*OMe)PhSCF_2_CH_3_, then the resultant (demethylated) phenol sulfoxide had an enantiomeric excess of 60%, and when the substrate was the β-substituted-SCF_2_CH_3_ naphthalene, then the enantiomeric excess of the resultant sulfoxide was 54%. There was no evidence of defluorination, unlike the corresponding oxygen ether (*p-*OMe)PhOCF_2_CH_3_, which was converted to the (demethylated) phenol acetate ester during *C. elegans* incubation. We conclude that the aryl–S–CF_2_CH_3_ motif is metabolised in a similar manner to aryl–SCF_3_, a motif that is being widely explored in discovery chemistry. It is however, significantly less lipophilic than aryl-SCF_3_ which may offer a practical advantage in tuning overall pharmacokinetic profiles of molecules in development.

## Introduction

**Figure 1:**
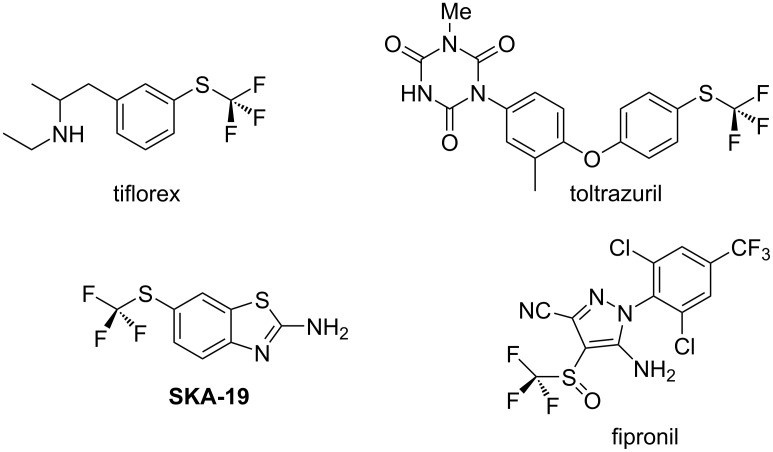
Structures of trifluoromethyl sulfonyl ether bioactives.

Fluorine and fluorinated substituents are routinely used to modify the properties of lead compounds in medicinal chemistry and in bioactive discovery programmes [1–2]. To this end, aryl–F and aryl–CF_3_ are the most common modifications found in compounds registered in the patent literature [3], substituents which are typically introduced to stop metabolism of aryl rings [4]. Other fluorinated motifs are gaining in importance too, such as aryl–OCF_3_ and aryl–SCF_3_ ethers, although these substituents can significantly raise lipophilicity (log P) [5–6]. There are relatively few bioactives on the market in this class, some of which are illustrated in Figure 1, however, it is noticeable that there is an increasingly active methodology focus describing new ways to introduce -OCF_3_ and -SCF_3_, and it can be anticipated that the number of bioactives of this class will increase [7]. The -OCF_3_ substituent is not directly metabolised, however, the sulphur associated with the -SCF_3_ group is susceptible to in vivo oxidation.

**Figure 2:**
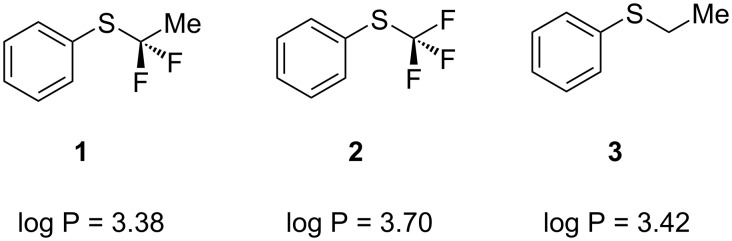
Comparison of log P values of comparative aryl thioether motifs [18].

Tiflorex, an appetite suppressant, toltrazuril, a coccidiostat used as an additive to poultry feed, and SKA-19, an anticonvulsant as shown in Figure 1, are three such compounds that are of commercial significance [8–10]. Metabolism studies in both of these cases show that the major metabolites are their corresponding sulfoxides (Ar–S(O)CF_3_) and sulfones (Ar–S(O)_2_CF_3_) [8–9]. Indeed, in the case of the insecticide fipronil it is actually the sulfoxide (Ar–S(O)CF_3_) that is marketed as the active component [11–12]. The increase in lipophilicity associated with these substituents is not always desirable. In this regard, partially fluorinated alkyl substituents become an interesting alternative, as the fluorines polarise the adjacent hydrogens and lipophilicity reduces relative to the perfluoro substituents [13–15]. In this context we recently introduced aryl α,α-difluoroethyl thioethers such as **1** as a motif of this class [16–18]. Log P assessments of PhSCF_2_CH_3_ (**1**) indicate that it is more polar than the PhSCF_3_ (**2**) and also the aliphatic PhSCH_2_CH_3_ ether **3** as shown in Figure 2. Therefore, there is potential for the inclusion of this motif in candidate molecules without a significant increase in log P.

Having explored synthetic routes and log P evaluations, we now report our initial studies on the metabolism of the ArSCF_2_CH_3_ substituent. In this context, the potential for P450 oxidation at sulphur is the most obvious metabolic vulnerability, and also hydrolytic susceptibility to release fluoride. We chose to explore the metabolism of aryl α,α-difluoroethyl thioethers **4** and **5** by *Cunninghamella elegans* as representative compounds of this class. This fungus is rich in cytochrome P450 activity and has been used as a model organism in which to mimic phase one mammalian metabolism of xenobiotics [19–21].

## Results and Discussion

**Figure 3:**
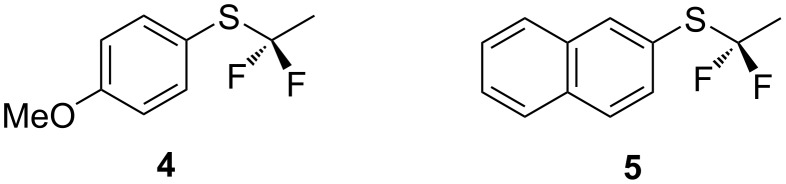
α,α-Difluoroethyl thioether substrates for metabolism studies.

The two aryl α,α-difluoroethyl thioethers **4** and **5** in Figure 3 were selected for *C. elegans* incubations rather than **1**, as they are less volatile, to avoid evaporation losses during extended incubations and work-up. Cultures of *C. elegans* were grown in Saboraud dextrose medium and incubated on an orbital shaker at 28 °C for 72 hours. New metabolites could be conveniently observed by extracting aliquots of the media into diethyl ether and dichloromethane (DCM) and then carrying out HPLC analyses.

**Scheme 1:**
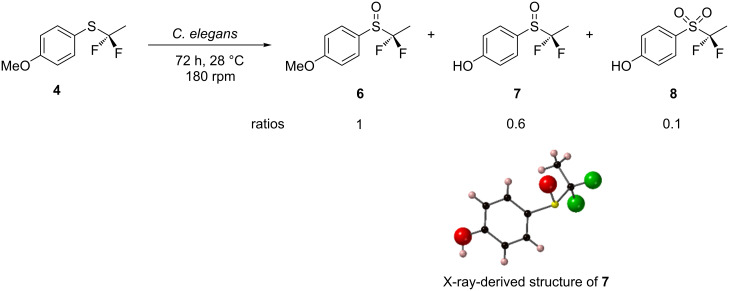
Fluorometabolites **6**–**8** isolated after incubation of **4** with *C. elegans*. Ratios are the average of three incubations. The structure of **7** was confirmed by X-ray structure analysis.

Incubation of thioether **4** with *C. elegans* led to the identification of three metabolites **6**–**8** as illustrated in Scheme 1. These were isolated by semi-preparative HPLC. Two of these (**6** and **7**) displayed an AB system in the ^19^F NMR spectrum consistent with non-equivalence of the fluorines, immediately indicative of sulfoxide formation. Isolation and subsequent ^1^H and ^19^F NMR analyses as well as high-resolution mass spectrometry secured the identity of these metabolites as sulfoxides **6** and **7** and sulfone **8**. The structure of sulfoxide **7** was also confirmed by X-ray structure analysis. The incubation of **4** in *C. elegans*, and then HPLC purification, was repeated three times all with very similar results. These incubations showed full conversion into the metabolites, and no starting thioether **4** was observed by NMR or HPLC.

Sulfone **8** was a relatively minor metabolite, at only 10% of sulfoxide **6**. A chiral HPLC (IC column, solvent: 5% isopropanol in hexane; 1 mL/min) enantiomeric assay was conducted for sulfoxide **6** and the outcome compared with a racemic sample of **6**, prepared by chemical oxidation of thioether **4** [22]. Although we could not determine the absolute stereochemistry of the predominant enantiomer, it was clear from the assay that there was a significant enantiomeric ratio (4:1) of **6** which translates to a 60% enantiomeric excess (ee, see Supporting Information File 1). We note that there have been chemical methods developed for the synthesis of enantiomerically enriched aryl fluoroalkyl sulfoxides, however, this appears to be the first enzymatic approach [23–25].

**Scheme 2:**
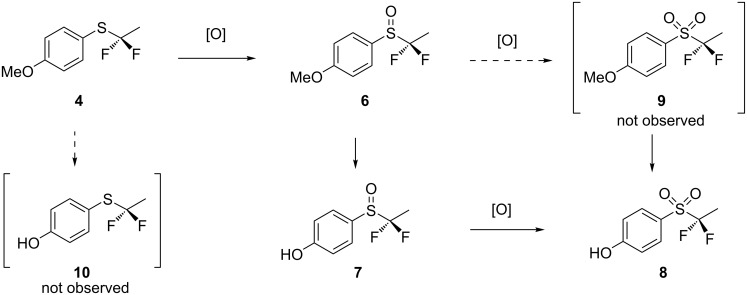
Putative pathways for (α,α-difluoroethyl)(4-methoxyphenyl)sulfane (**4**) metabolism.

Although the fungus was able to both oxidise the sulfur and demethylate the *para*-methoxy group of **4**, there was no obvious presence of novel metabolites which did not contain fluorine. This points to a hydrolytically stable motif over the period of the incubation. In an effort to establish the sequence of events through which these metabolites are generated, each was separately re-incubated with *C. elegans* to establish if they could be further metabolised. Product profiles were again determined by HPLC analysis and relationships are summarised in Scheme 2.

Incubation of racemic sulfoxide **6** led to a similar outcome to that for **4** with the formation of phenol sulfoxide **7** and phenol sulfone **8** suggesting that sulfoxide **6** is the first formed metabolite with demethylation following subsequently at a slower rate. The experiments on the re-incubation of phenols **7** and **8** failed to lead to any further metabolism, where they appeared to be stable metabolites, certainly for the period (72 h) of the incubation. We were unable to observe a transformation from sulfoxide **6** to sulfone **9** in these re-incubation experiments. It may be that there is a barrier to uptake of the phenols into the fungal cells and that they are actively exuded when generated within the cell. As a final experiment sulfone **9**, which was not observed as a metabolite, was prepared by *m*CPBA oxidation of thioether **4**. This sulfone was then incubated with *C. elegans* and it gave rise to demethylated phenol **8** as the sole metabolite, consistent with an active demethylation capacity of this fungus. Non-oxidised phenol **10** was also not observed in this study. Overall this suggests that sulfoxidation is very active and outcompetes demethylation, however, that demethylation is significantly more active that the second oxidation of sulfoxides to sulfones.

**Scheme 3:**
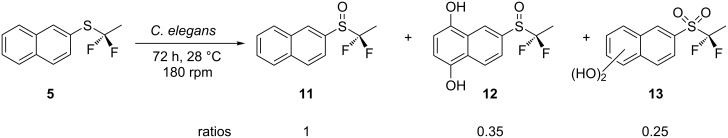
*C. elegans* incubation of **5**. Ratios are the average of three incubations.

Incubation of naphthalene **5** with *C. elegans*, generated three new metabolites **11**–**13** which arose by oxidations at sulphur and hydroxylations of the naphthalene ring, as summarised in Scheme 3. These metabolites were isolated by reversed-phase HPLC and characterised by ^1^H and ^19^F NMR and mass spectrometry. This identified sulfoxides **11** and **12** as the major metabolites and a trace amount of a minor sulfone which had the general structure **13** as determined by mass spectrometry. Naphthalene **5** was fully converted. Again, initial sulfoxidation dominates, but the second oxidation of the sulfoxide to a sulfone appears to be a slower process, and is outcompeted by aryl hydroxylation reactions. The experiment was conducted three times under similar conditions, all with very similar outcomes.

In order to explore the stereoselectivity of this sulfoxidation, sulfoxide **11** isolated from the *C. elegans* incubation was analysed by chiral HPLC (IC column, solvent: 5% isopropanol in hexane; 1 mL/min). This was compared to a racemic reference sample of **11** prepared by chemical oxidation of thioether **5**. The resultant enantiomeric ratio for **11** was determined to be 3.3:1, which translates to a 54% ee (absolute stereochemistry not determined). Again, the sulfoxidation shows a significant stereochemical bias.

**Scheme 4:**
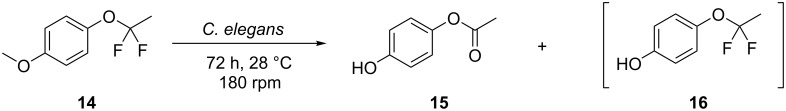
Incubation (four times) of oxygen ether **14** with *C. elegans*, gave 4-acetoxyphenol (**15**) as the major metabolite. On one occasion **16** was tentatively observed as a minor metabolite.

The stability of the aryl thioethers can be contrasted with the hydrolytic lability of the analogous oxygen ethers. By way of example α,α-difluoroethyl ether **14** [18] was incubated with cultures of *C. elegans* under the standardised conditions, as illustrated in Scheme 4. ^19^F NMR of the extract indicated a trace of residual starting material with one major metabolite which was isolated by HPLC. This was identified as 4-acetoxyphenol (**15**). Aryl ether demethylation remains highly active, but unlike the α,α-difluoroethyl thioethers **4** and **5**, the oxygen ether is vulnerable to hydrolytic fluoride release. Oxygen ether **14** is particularly labile, and the incubation proceeds to full conversion to generate acetate ester **15**.

The incubation of **14** was carried out four times, all affording consistent results and generating **15** as the clearly identifiable product. However, in one experiment minor traces of a fluorinated metabolite was observed. Although a full characterisation was not possible due to the low levels recovered, ^1^H and ^19^F NMR for this metabolite showed an intact -OCF_2_CH_3_ motif, as well as a *para-*substitution pattern, which suggested the identity of 4-(1,1-difluoroethoxy)phenol (**16**).

## Conclusion

In conclusion, we have explored *Cunninghamella elegans* fungal metabolism of the α,α-difluoroethyl thioether (Ar–SCF_2_CH_3_) motif, which we introduced recently as a more polar alternative to RSCF_3_. It emerges to have a similar metabolism to ArSCF_3_ in that it progresses to the sulfoxide and then the sulfone, although in this study the initial oxidation to the sulfoxide was significantly more rapid than the second oxidation to the sulfone. The first oxidation gave enantiomerically enriched sulfoxides (Ar–S(O)CF_2_CH_3_) in the 54–60% ee range. This could arise by the action of more than one P450 enzyme. There was no evidence of defluorination, or hydroxylation at the terminal -CH_3_ group. The corresponding oxygen ether **14** was susceptible to hydrolytic defluorination to generate an acetate ester. The α,α-difluoroethyl thioether (Ar–SCF_2_CH_3_) motif can be readily prepared from thiols and emerges as a potentially attractive substituent for consideration by medicinal chemists and for other areas of bioactives discovery research, to complement the widely used ROCF_3_ and RSCF_3_ groups and there is a clear similarity to the ‘polar lipophilic’ groups ROCF_2_H, and RSCF_2_H [26].

## Experimental

### Microorganism growth

*Cunninghamella elegans* DSM1908 was grown on Saboraud dextrose agar gel plates for 120 h at 28 °C, from previous stocks. The plates were stored at 4 °C for a maximum of 4 months. Liquid cultures were prepared by inoculation from mycelium from the plates, into Saboraud dextrose broth (50 mL), and grown on an incubator shaker for 72 h at 28 °C and 180 rpm.

### Synthesis of substrates

Thioethers **4** and **5** and oxygen ether **14** were prepared as previously described [18]. Compounds **6**, **8** and**11** were prepared as described below.

#### 1-(α,α-Difluoroethyl)sulfinyl)-4-methoxybenzene (**6**)

(1,1-Difluoroethyl)(4-methoxyphenyl)sulfane (**4**, 5 mg, 0.025 mmol) was added to a round bottom flask with a stirring bar and dissolved in a mixture of DCM (3 mL) and methanol (0.3 mL). The solution was stirred at room temperature until homogenisation (5 min). AlCl_3_ (1.8 mg, 0.012 mmol) was added, and the solution stirred for 5 min, prior to the addition of [bis(acetoxy)iodo]benzene (BAIB, 7.3 mg, 0.025 mmol). The reaction was left to stir overnight. After 16 h, the solvents were evaporated under reduced pressure. The remaining mixture showed the formation of **6** with 66% conversion from the starting material **4**. Further purification was achieved by reversed-phase HPLC in a Phenomenex Luna SP column, with 60:40 AcCN/water (supplemented with 0.05% TFA) at a flow rate of 1 mL/min. The product **6** was isolated at *t*_R_ = 24 min, which was consistent with the metabolic experiment’s data. ^1^H NMR (500 MHz, chloroform-*d*) δ_H_ 7.65 (d, *J* = 8.9 Hz, 2H), 7.08 (d, *J* = 8.9 Hz, 2H), 3.89 (s, 1H), 1.81 (t, *J* = 18.4 Hz, 1H); ^19^F NMR (471 MHz, chloroform-*d*) δ_F_ −93.4 (d, *J* = 225.1 Hz), −97.1 (d, *J* = 225.1 Hz).

#### 1–(α,α-Difluoroethyl)sulfonyl)-4-hydroxybenzene (**8**)

(1,1-Difluoroethyl)(4-methoxyphenyl)sulfane (**4,** 5 mg, 0.025 mmol) was added to a round bottom flask containing a stirring bar, and dissolved in CH_2_Cl_2_ (1.5 mL). *m*CPBA was added to the solution (21 mg, 0.122 mmol), and the mixture was stirred at rt overnight. The reaction was quenched by addition of a saturated solution of NaHCO_3_. The aqueous phase was extracted into CH_2_Cl_2_ (3 × 3 mL). The combined organic phases were combined, dried over Na_2_SO_4_, filtered and concentrated under reduced pressure, yielding to **8** with 100% conversion. Further purification was carried out by column chromatography, starting with 100% petroleum ether, followed by 15% EtOAc in petroleum ether, affording **8** in quantitative yield. ^1^H NMR (500 MHz, chloroform-*d*) δ_H_ 7.84 (d, *J* = 8.6 Hz, 2H), 7.02 (d, *J* = 8.6 Hz, 2H), 3.91 (s, 3H), 2.02 (t, *J* = 18.3 Hz, 3H); ^19^F NMR (471 MHz, chloroform-*d*) δ_F_ −97.3 (s); ^13^C NMR (126 MHz, chloroform-*d*) δ_C_ 165.2, 133.1, 122.9, 114.7, 55.8, 16.6 (t, *J* = 22.2 Hz); HMRS (ESI) *m*/*z*: [M + H]^+^ calcd for C_9_H_11_F_2_O_3_S, 236.0310; found, 236.0390; [M + Na]^+^ calcd, 259.0211; found, 259.0216.

#### 2–(α,α-Difluoroethyl)sulfinyl)naphthalene (**11**)

(1,1-Difluoroethyl)(naphthalene-2-yl)sulfane (**5**, 5 mg, 0.022 mmol) was added to a round bottom flask with a stirring bar and dissolved in a mixture of DCM (3 mL) and methanol (0.3 mL). The solution was stirred at room temperature until homogenisation (5 min). AlCl_3_ (1.5 mg, 0.011 mmol) was added, and the solution stirred for 5 min, prior to the addition of [bis(acetoxy)iodo]benzene (BAIB, 11.1 mg, 0.022 mmol). The reaction was left to stir overnight. After 16 h, the solvents were evaporated under reduced pressure. Further purification was achieved by reversed-phase HPLC in a Phenomenex Luna SP column, with 60:40 AcCN/water (supplemented with 0.05% TFA) at a flow rate of 1 mL/min, which afforded **11** in 30% yield. The product **11** was isolated at *t*_R_ = 37 min, which was consistent with the metabolic experiments’ data. ^1^H NMR (500 MHz, chloroform-*d*) δ_H_ 8.28 (s, 1H), 8.01 (d, *J* = 8.7 Hz, 1H), 7.99–7.92 (m, 2H), 7.69 (ddt, *J* = 8.7, 2.6, 1.3 Hz, 1H), 7.67–7.60 (m, 2H), 1.77 (t, *J* = 18.5 Hz, 3H); ^19^F NMR (471 MHz, chloroform-*d*) δ_F_ −92.9 (d, *J* = 227.0 Hz), −96.0 (d, *J* = 227.0 Hz); ^13^C NMR (126 MHz, chloroform-*d*) δ_C_ 145.9 (*C*-Ar, visible in HMBC), 135.1 (s, *C*-Ar), 133.6 (t, *J* = 218.8 Hz, *C*F_2_), 129.3 (s, *C*-Ar), 128.8 (s, *C*-Ar), 128.5 (s, *C*-Ar), 128.1 (s, *C*-Ar), 127.5 (s, *C*-Ar), 126.8 (s, *C*-Ar), 121.0 (s, *C*-Ar), 111.7 (*C*-Ar, visible in HMBC), 16.5 (t, *J* = 22.1 Hz, CF_2_*C*H_3_); HRMS (ESI^+^) *m*/*z*: [M + H]^+^ calcd for C_12_H_11_OF_2_S, 241.0420; found, 241.0491.

### Biotransformations and extraction conditions

Culture media was purchased from Sigma-Aldrich. Each thioether (5–10 mg) was dissolved in DMF (50 µL) and inoculated into cultures of *C. elegans*. The cultures were incubated at 28 °C and 180 rpm for 72 h. Blank experiments were carried out in the absence of *C. elegans*. After 72 h, the fungal biomass was removed and washed with diethyl ether. The supernatant was extracted into diethyl ether (3 × 50 mL) and dichloromethane (3 × 50 mL), and the combined organic extracts were dried over anhydrous Na_2_SO_4_, filtered and evaporated under reduced pressure. The extracts were analysed by ^1^H and ^19^F NMR before further purification by HPLC.

### Purification of the fluorometabolites

The fluorometabolites (sulfoxides and sulfones) were isolated by reversed-phase HPLC using a Shimadzu Prominence (SIL-20A HT autosampler, CL-20AT ternary pump, DGU-20A3R solvent degasser, SPD 20A UV detector and CVM-20A controller module), equipped with a Phenomenex semi-preparative Luna C18 column. Purification of the metabolites was carried out by HPLC, using an eluent system of 60:40 AcCN/water (both supplemented with 0.05% TFA), at a flow rate of 1 mL/min. For this, the extracts were redissolved in AcCN (1 mL, HPLC grade), and injected in 10–20 µL batches. Each fluorometabolite was separated in vials, evaporated and analysed.

Structural analysis of the resulting metabolites and remaining starting materials was carried out by NMR characterisation (^1^H, ^19^F, ^13^C, COSY, HSQC and HMBC) and accurate mass spectrometry. ^19^F{^1^H} and ^13^C{^1^H}) NMR spectra were recorded on Bruker Avance III 500 or Bruker Avance III 500 HD spectrometers (500 MHz ^1^H, 476 MHz ^19^F, 126 MHz ^13^C). High-resolution mass spectrometry was acquired using electrospray ionisation (ESI), on a ThermoFisher Excalibur Orbitrap Spectrometer, operating in positive and negative mode, from solutions of the analyte in methanol or acetonitrile.

The X-ray structure analysis of **7** (CCDC deposition code 1911894) was obtained using a Rigaku XtaLAB P200 diffractometer, using multi-layer mirror monochromed Mo Kα radiation.

## Supporting Information

File 1 Further details of equipment specifications and compound characterisation.
