# Supplementary material for: Fluorine-containing substituents: metabolism of the α,α-difluoroethyl thioether motif
Source: Beilstein J Org Chem. 2019 Jun 28;15:1441–7. doi: 10.3762/bjoc.15.144 (PMC6604701; doi:10.3762/bjoc.15.144)
Supplement: File 1 — Further details of equipment specifications and compound characterisation. [file Beilstein_J_Org_Chem-15-1441-s001.pdf]

BEILSTEIN JOURNAL OF ORGANIC CHEMISTRY

Supporting Information

for

Fluorine-containing substituents: metabolism of the α,α-difluoroethyl thioether motif

Andrea Rodil, Alexandra M. Z. Slawin, Nawaf Al-Maharik, Ren Tomita and David O'Hagan

Beilstein J. Org. Chem. **2019,** *15,* 1441–1447. doi:10.3762/bjoc.15.144

Further details of equipment specifications and compound characterisation

License and Terms: This is a supporting information file under the terms of the Creative Commons Attribution License (http://creativecommons.org/licenses/by/4.0). Please note that the reuse, redistribution and reproduction in particular requires that the authors and source are credited.
The license is subject to the *Beilstein Journal of Organic Chemistry* terms and conditions: (https://www.beilstein-journals.org/bjoc)

Contents

1. General procedures and methods S2

2. Experimental general procedures for metabolism studies S3

2.1 Preparation of cultures and inoculation of xenobiotics S3

2.2 Extraction and purification of the metabolites S3

3. Thio- and oxyether metabolism S3

3.1 (1,1-Difluoroethyl)(4-methoxyphenyl)sulfane (**4**) S3

3.2 (1,1-Difluoroethyl)(naphthalene-2-yl)sulfane (**5**) S5

3.3 1-(1,1-Difluoroethoxy)-4-methoxybenzene (**14**) S7

4. Synthesis of racemic materials for enantiomeric excess analysis and further characterisation of metabolites S8

4.1 1-((1,1-Difluoroethyl)sulfonyl)-4-methoxybenzene (**8**) S8

4.2 Racemic 1-((1,1-difluoroethyl)sulfinyl)-4-methoxybenzene (**6**) S8

4.3 Racemic 2-((1,1-difluoroethyl)sulfinyl)naphthalene (**11**) S9

5. Enantiomeric excess analysis S10

5.1 1-((1,1-Difluoroethyl)sulfinyl)-4-methoxybenzene (**6**) S10

5.2 2-((1,1-Difluoroethyl)sulfinyl)naphthalene (**11**) S11

6. Single crystal X-ray analysis S12

7. References S20

1. General procedures and methods

All the thioethers, sulfones and sulfoxides used were synthesised in the laboratory of Prof. David O'Hagan, either as published before (Tomita et al., 2018) or as detailed in the following sections. The rest of the chemicals, media and materials were bought from Sigma-Aldrich or Alfa Aesar. Fungus *Cunninghamella elegans* was originally donated by Dr. Cormac Murphy (University College Dublin, Dublin, Ireland), and stored as an agar gel at 4 °C, from which our current agar gels were prepared.

All the glassware, materials and media used for microbiological purposes were sterilised by autoclaving prior to their use. The aseptic conditions were maintained during the preparation, growth and incubation of the fungal cultures.

All the glassware used for chemical synthesis was oven-dried, cooled and used under nitrogen atmosphere, unless otherwise stated.

The progress of reactions was followed by thin-layer chromatography (TLC), using aluminium plates coated with silica gel ($60F_{245}$ Merck). The TLC plates were examined under UV light at 254 and 266 nm, before being visualised with alkaline potassium permanganate.

Crude extracts were analysed by ^{1}H and ^{19}F NMR. Proton and proton decoupled ($^{19}F\{^{1}H\}$, $^{13}C\{^{1}H\}$) nuclear magnetic resonance spectra were recorded on Bruker Avance III 500 or Bruker Avance III 500 HD spectrometers (500 MHz ^{1}H, 476 MHz ^{19}F, 126 MHz ^{13}C). Bidimensional correlation spectra were also analysed for the correct assignment of signals. Chemical shifts (δ) are expressed in ppm, and quoted relative to the residual solvent signal. Proton coupling constants (J) are given in Hz, and quoted to the nearest 0.1 Hz. Identical coupling constants are averaged.

The fluorometabolites (sulfoxides and sulfones) were isolated using a Shimadzu Prominence (SIL-20A HT autosampler, CL-20AT ternary pump, DGU-20A3R solvent degasser, SPD 20A UV detector and CVM-20A controller module), equipped with a Phenomenex semi-preparative Luna C_{18} column. The AcCN and water eluents used for HPLC were filtered and supplemented with 0.05% TFA.

High-resolution mass spectrometry was acquired using electrospray ionisation (ESI), on a ThermoFisher Excalibur Orbitrap Spectrometer, operating in positive and negative mode, from solutions of the analyate in methanol or acetonitrile. Mass analysis was done at the University of St Andrews Mass Spectrometry facility by Mrs. Caroline Horsburgh. Mass units are reported in Daltons (Da).

X-ray crystal structures were obtained on a Rigaku XtaLAB P200 diffractometer, using multi-layer mirror monochromed Mo-Kα radiation, by Prof. Alexandra Slawin (University of St Andrews). The data was analysed using CrystalMaker.

2. Experimental general procedures for metabolism studies

2.1 Preparation of cultures and inoculation of xenobiotics

Sterile Saboraud Dextrose Medium (SBD, 50 mL) was inoculated with a piece of fungal agar plate (1 cm x 1 cm) at room temperature. The cultures were left to grow for 72 h at 28 °C and rotary agitation (180 rpm). After 72 h, the corresponding thioethers were added dissolved in DMF solution (5–10 mg in 50 µL) to the grown cultures, and left to incubate for further 72 h at 28 °C and 180 rpm.

2.2 Extraction and purification of the metabolites

After incubation, the fungal biomass was separated from the liquid culture, and the supernatant was extracted with ethyl ether (3 × 50 mL) and DCM (3 × 50 mL). The combined organic phases were dried over anhydrous Na_2SO_4, filtered and concentrated under reduced pressure. The extracts were analysed by ^{1}H and ^{19}F NMR before further purification.

Purification of the metabolites was carried out by reversed-phase HPLC, using an eluent system of 60:40 AcCN/water (both supplemented with 0.05% TFA), at a flow rate of 1 mL/min. Structural analysis of the resulting metabolites and remaining starting materials was carried out by full NMR characterisation (^{1}H, ^{19}F, ^{13}C, COSY, HSQC and HMBC) and accurate mass spectrometry. X-ray structures were obtained when possible.

3. Thio- and oxyether metabolism

3.1 (1,1-Difluoroethyl)(4-methoxyphenyl)sulfane (4)

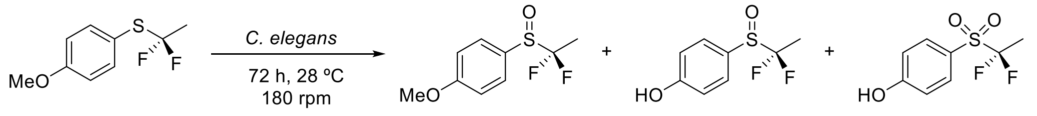

(1,1-Difluoroethyl)(4-methoxyphenyl)sulfane (**4**) was dissolved in DMF (50 µL) and added to a mature culture of *C. elegans*. The compound was left to incubate with the fungus for 72 h. at 28 ºC and 180 rpm. After 72 h, the fungus was centrifuged down, and the supernatant was extracted with DCM (3 x 50 mL) and Et_2O (3 × 50 mL). The combined organic phases were dried over Na_2SO_4, filtered and evaporated under reduced pressure. Purification of the metabolite and residual starting material was achieved by reversed-phase HPLC, in a Phenomenex Luna, and eluting with a mixture of 60:40 AcCN:water, both supplemented with 0.05% of TFA, at a flow rate of 1 mL/min. This afforded **6** at a t_R = 24 min, **7** at a t_R = 16 min and **8** at a t_R = 21 min.

1-((1,1-Difluoroethyl)sulfinyl)-4-methoxybenzene (6)

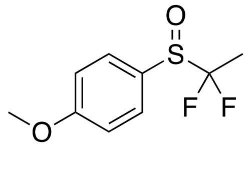

^{1}H NMR (500 MHz, chloroform-*d*) δ_H 7.64 (2H, d, *J* = 8.9 Hz, *H*-Ar), 7.06 (2H, d, *J* = 8.9 Hz, *H*-Ar), 3.88 (3H, s, OC*H*3), 1.75 (3H, t, *J* = 18.4 Hz, CF_2CH_3); **^{19}F{^{1}H} NMR** (470 MHz, chloroform-*d*) δ_F -94.2 (d, *J* = 225.8 Hz), - 97.6 (d, *J* = 225.8 Hz); **^{13}C NMR** (126 MHz, chloroform-*d*) δ_C 163.1 (s, *C*-4), 128.1 (t, *J* = 323.8 Hz, CF_2), 127.6 (s, *C*-2), 114.8 (s, *C*-3), 55.6 (s, OCH_3), 16.5 (t, *J*= 22.3 Hz, CF_2CH_3); **HRMS** (ESI$^+$) *m/z* calc. for $C_9H_{11}O_2F_2S$ $[M+H]^+$ 221.0448, found 221.0440.

4-((1,1-Difluoroethyl)sulfinyl)phenol (7)

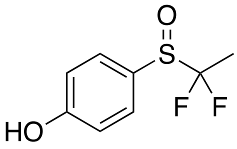

^{1}H NMR (500 MHz, chloroform-*d*) δ_H 7.60 (2H, d, *J* = 8.9 Hz, *H*-Ar), 7.01 (2H, d, *J* = 8.9 Hz, *H*-Ar), 1.75 (3H, t, *J* = 18.4 Hz, CF_2CH_3); **^{19}F{^{1}H} NMR** (470 MHz, chloroform-*d*) δ_F -94.1 (d, *J* = 225.1 Hz), - 97.6 (d, *J* = 225.1 Hz); **^{13}C NMR** (126 MHz, chloroform-*d*) δ_C 159.7 (s, *C*-4), 127.9 (s, *C*-2), 125.4 (t, *J* = 276.2 Hz, CF_2, visible in HMBC), 116.4 (s, *C*-3), 16.5 (t, *J*= 22.3 Hz, CF_2CH_3); **HRMS** (ESI$^+$) *m/z* calc. for $C_8H_9O_2F_2S$ $[M+H]^+$ 207.0291, found 207.0286.

4-((1,1-Difluoroethyl)sulfonyl)phenol (8)

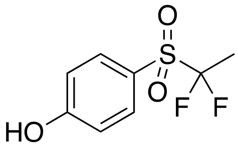

^{1}H NMR (500 MHz, chloroform-*d*) δ_H 7.87 (2H, d, *J* = 8.9 Hz, *H*-Ar), 7.01 (2H, d, *J* = 8.9 Hz, *H*-Ar), 2.02 (3H, t, *J* = 18.3 Hz, CF_2CH_3); **^{19}F{^{1}H} NMR** (470 MHz, chloroform-*d*) δ_F -97.2 (s); **^{13}C NMR** (126 MHz, chloroform-*d*) δ_C 161.8 (s, *C*-4), 133.5 (s, *C*-3), 123.6 (t, *J* = 262.9 Hz, *C*F_2, visible in HMBC), 116.3 (s, *C*-2), 16.6 (t, *J* = 22.4 Hz, CF_2CH_3); **HRMS** (ESI$^+$) *m/z* calc. for $C_8H_8O_3F_2SNa$ $[M+Na]^+$ 245.0060, found 245.0055; **HRMS** (ESI$^-$) *m/z* calc. for $C_8H_7O_3F_2S$ $[M-H]^-$ 221.0084, found 221.0087.

Reproducibility of results

Structure	Incubation Number	Ratio by NMR	Ratio by HPLC
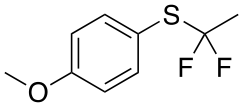	1	-	-
	2	-	-
	3	-	-
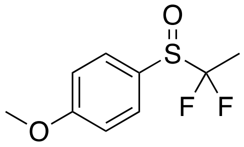	1	1	1
	2	1	1
	3	1	1
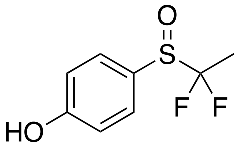	1	0.6	0.6
	2	0.8	0.7
	3	0.8	0.6
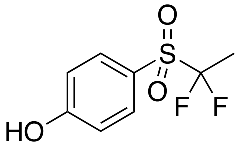	1	0.1	0.1
	2	0.1	0.1
	3	0.15	0.1

3.2 (1,1-Difluoroethyl)(naphthalene-2-yl)sulfane (5)

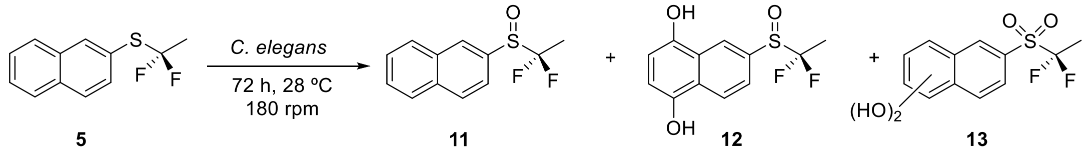

(1,1-Difluoroethyl)(naphthalene-2-yl)sulfane (**5**) was dissolved in DMF (50 µL) and added to a mature culture of *C. elegans*. The compound was left to incubate with the fungus for 72 h. at 28 ºC and 180 rpm. After 72 h, the fungus was centrifuged down, and the supernatant was extracted with DCM (3 × 50 mL) and Et_2O (3 × 50 mL). The combined organic phases were dried over Na_2SO_4, filtered and evaporated under reduced pressure. Purification of the metabolite and residual starting material was achieved by reversed-phase HPLC, in a Phenomenex Luna, and eluting with a mixture of 60:40 AcCN:water, both supplemented with 0.05% of TFA, at a flow rate of 1 mL/min. This afforded **11** at a t_R = 37 min, **12** at a t_R = 13 min and **13** at a t_R = 17 min.

2-((1,1-Difluoroethyl)sulfinyl)naphthalene (11)

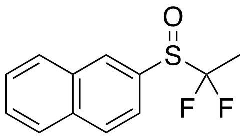

^{1}H NMR (500 MHz, chloroform-*d*): δ_H 8.28 (s, 1H), 8.01 (d, *J* = 8.7 Hz, 1H), 7.99 – 7.92 (m, 2H), 7.69 (ddt, *J* = 8.7, 2.6, 1.3 Hz, 1H), 7.67 – 7.60 (m, 2H), 1.77 (t, *J* = 18.5 Hz, 3H); **^{19}F NMR** (471 MHz, chloroform-*d*): δ_F -92.9 (d, *J* = 227.0 Hz), -95.6 (d, *J* = 227.0 Hz); **HRMS** (ESI$^+$) *m/z* calc. for $C_{12}H_{11}OF_2S$ $[M+H]^+$ 241.0420, found 241.0491. Data is consistent with the full characterisation carried out for **11** from chemical reaction.

6-((1,1-Difluoroethyl)sulfinyl)naphthalene-1,4-diol (12)

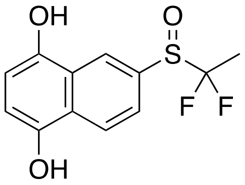

^{1}H NMR (500 MHz, chloroform-*d*): δ_H 7.81 (1H, d, *J* = 7.9 Hz, *H*-Ar), 7.61 (1H, d, *J* = 7.9 Hz, *H*-Ar), 7.44 (1H, s, *H*-Ar), 6.50 (1H, d, *J* = 11.0 Hz, *H*-Ar), 6.12 (1H, d, *J* = 11.0 Hz, *H*-Ar), 1.80 (3H, t, *J* = 18.4 Hz, CF_2CH_3); **^{19}F NMR** (471 MHz, chloroform-*d*): δ_F -93.2(d, *J* = 226.9 Hz), -96.7 (d, *J* = 226.9 Hz); **HRMS** (ESI$^-$) *m/z* calc. for $C_{12}H_9O_3F_2S$ $[M-H]^-$ 271.0246, found 271.0245.

General (1,1-difluoroethyl)sulfinyl)naphthalene (13)

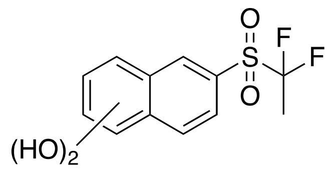

^{19}F NMR (471 MHz, chloroform-*d*): δ_F -96.8 (s); ***m/z*** calc. for $C_{12}H_{10}O_4F_2S$ $[M]^+$ 288, found 288.

Reproducibility of results

Structure	Incubation Number	Ratio by NMR	Ratio by HPLC
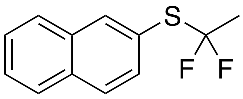	1	-	-
	2	-	-
	3	-	-
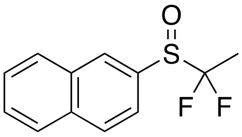	1	1	1
	2	1	1
	3	1	1
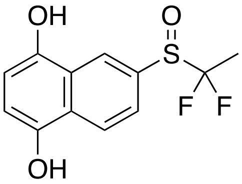	1	0.35	0.30
	2	0.40	0.30
	3	0.35	0.35
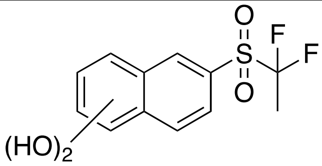	1	0.25	0.05
	2	0.26	0.05
	3	0.29	0.06

3.3 1-(1,1-Difluoroethoxy)-4-methoxybenzene (14)

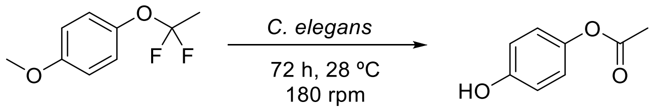

14 **15**

1-(1,1-Difluoroethoxy)-4-methoxybenzene (**14**) was dissolved in DMF (50 µL) and added to a mature culture of *C. elegans*. The compound was left to incubate with the fungus for 72 h. at 28 °C and 180 rpm. After 72 h, the fungus was centrifuged down, and the supernatant was extracted with EtOAc (3 × 50 mL). The combined organic phases were dried over Na_2SO_4, filtered and evaporated under reduced pressure. Purification of the metabolite and residual starting material was achieved by reversed-phase HPLC, in a Phenomenex Luna, and eluting with a mixture of 60:40 AcCN/water, both supplemented with 0.05% of TFA, at a flow rate of 1 mL/min. This afforded **15** at a t_R = 25 min.

4-Acetoxyphenol (15)

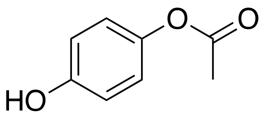

^{1}H NMR (500 MHz, chloroform-*d*) δ_H 6.95 (2H, d, *J* = 8.9 Hz, *H*-Ar), 6.82 (2H, d, *J* = 8.9 Hz, *H*-Ar), 2.28 (3H, t, *J* = 18.4 Hz, COCH_3); **^{13}C NMR** (126 MHz, chloroform-*d*) δ_C 160.8 (s, *C*O, visible in HMBC), 122.7 (s, *C*-Ar), 116.1 (s, *C*-Ar), 21.1 (s, COCH_3). Data is in good agreement with the literature values (Coombes et al., 2008).

4-(1,1-Difluoro)ethoxy phenol (16)

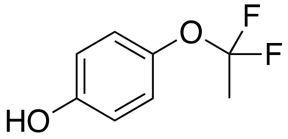

^{1}H NMR (500 MHz, chloroform-*d*) δ_H 6.75 (2H, d, *J* = 2.7 Hz, *H*-Ar), 6.64 (2H, d, *J* = 2.7Hz, *H*-Ar), 1.87 (3H, t, *J* = 13.3 Hz, CF_2CH_3); **^{19}F{^{1}H} NMR** (470 MHz, chloroform-*d*) δ_F -64.6 (s); **^{13}C NMR** (126 MHz, chloroform-*d*) δ_C 114.4 (s, *C*-Ar), 110.1 (s, *C*-Ar), 22.5 (t, *J* = 32.0 Hz, CF_2CH_3). Low amounts did not allow a full characterisation.

4. Synthesis of racemic materials for enantiomeric excess analysis and further characterisation of metabolites

4.1 1-((1,1-Difluoroethyl)sulfonyl)-4-methoxybenzene (9)

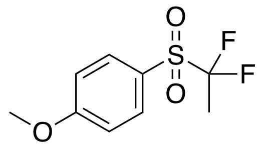

8

(1,1-Difluoroethyl)(4-methoxyphenyl)sulfane (**4**, 5 mg, 0.025 mmol) was added to a round bottom flask containing a stirring bar, and dissolved in CH_2Cl_2 (1.5 mL). *m*CPBA was added to the solution (21 mg, 0.122 mmol), and the mixture was stirred at r.t. overnight. The reaction was quenched by addition of a saturated solution of $NaHCO_3$. The aqueous phase was extracted with CH_2Cl_2 (3 × 3 mL). The combined organic phases were combined, dried over Na_2SO_4, filtered and concentrated under reduced pressure, yielding to **9** with 100% conversion. Further purification was carried out by column chromatography, starting with 100% petroleum ether, followed by 15% EtOAc in petroleum ether, affording **8** in quantitative yield. **^{1}H NMR** (500 MHz, chloroform-*d*): δ_H 7.84 (d, J = 8.6 Hz, 2H), 7.02 (d, J = 8.6 Hz, 2H), 3.91 (s, 3H), 2.02 (t, J = 18.3 Hz, 3H) **^{19}F NMR** (471 MHz, chloroform-*d*): δ_F -97.3 (s) **^{13}C NMR** (126 MHz, chloroform-*d*): δ_C 165.2, 133.1, 122.9, 114.7, 55.8, 16.6 (t, J = 22.2 Hz) **HMRS** (ESI) $C_9H_{11}F_2O_3S$ $[M+H]^+$ calculated for 236.0310, found 236.0390; $[M+Na]^+$ calculated for 259.0211, found 259.0216.

4.2 Racemic 1-((1,1-difluoroethyl)sulfinyl)-4-methoxybenzene (6)

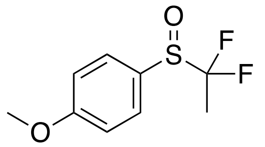

6

(1,1-Difluoroethyl)(4-methoxyphenyl)sulfane (**4**, 5 mg, 0.025 mmol) was added to a round bottom flask with a stirring bar and dissolved in a mixture of DCM (3 mL) and methanol (0.3 mL). The solution was stirred at room temperature until homogenisation (5 min). $AlCl_3$ (1.8 mg, 0.012 mmol) was added, and the solution stirred for 5 min, prior to the addition of BAIB ((bisacetoxyiodo)benzene, 7.3 mg, 0.025 mmol). The reaction was left to stir overnight. After 16 h, the solvents were evaporated under reduced pressure. The remaining mixture showed the formation of **6** with 66% conversion from the starting material **4**. Further purification was achieved by reversed-phase HPLC in a Phenomenex Luna SP column, with 60:40 AcCN/water (supplemented with 0.05% TFA) at a flow rate of 1 mL/min. The product **6** was isolated at t_R = 24 min, which was consistent with the metabolic experiment's data. **^{1}H NMR** (500 MHz, chloroform-*d*): δ_H 7.65 (d, J = 8.9 Hz, 2H), 7.08 (d, J = 8.9 Hz, 2H), 3.89 (s, 1H), 1.81 (t, J = 18.4 Hz, 1H) **^{19}F NMR** (471 MHz, chloroform-*d*): δ_F -93.4 (d, J = 225.1 Hz), -97.1 (d, J = 225.1 Hz).

4.3 Racemic 2-((1,1-difluoroethyl)sulfinyl)naphthalene (11)

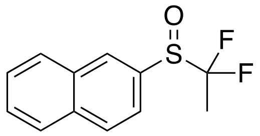

11

(1,1-Difluoroethyl)(naphthalene-2-yl)sulfane (**5**, 5 mg, 0.022 mmol) was added to a round bottom flask with a stirring bar and dissolved in a mixture of DCM (3 mL) and methanol (0.3 mL). The solution was stirred at room temperature until homogenisation (5 min). $AlCl_3$ (1.5 mg, 0.011 mmol) was added, and the solution stirred for 5 min, prior to the addition of BAIB (11.1 mg, 0.022 mmol). The reaction was left to stir overnight. After 16 h, the solvents were evaporated under reduced pressure. Further purification was achieved by reversed-phase HPLC in a Phenomenex Luna SP column, with 60:40 AcCN/water (supplemented with 0.05% TFA) at a flow rate of 1 mL/min, which afforded **11** in 30% yield. The product **11** was isolated at t_R = 37 min, which was consistent with the metabolic experiments' data. **^{1}H NMR** (500 MHz, chloroform-*d*): δ_H 8.28 (s, 1H), 8.01 (d, *J* = 8.7 Hz, 1H), 7.99 – 7.92 (m, 2H), 7.69 (ddt, *J* = 8.7, 2.6, 1.3 Hz, 1H), 7.67 – 7.60 (m, 2H), 1.77 (t, *J* = 18.5 Hz, 3H) **^{19}F NMR** (471 MHz, chloroform-*d*): δ_F -92.9 (d, *J* = 227.0 Hz), -96.0 (d, *J* = 227.0 Hz); **^{13}C NMR** (126 MHz, chloroform-*d*) δ_C 145.9 (*C*-Ar, visible in HMBC), 135.1 (s, *C*-Ar), 133.6 (t, *J* = 218.8 Hz, CF_2), 129.3 (s, *C*-Ar), 128.8 (s, *C*-Ar), 128.5 (s, *C*-Ar), 128.1 (s, *C*-Ar), 127.5 (s, *C*-Ar), 126.8 (s, *C*-Ar), 121.0 (s, *C*-Ar), 111.7 (*C*-Ar, visible in HMBC), 16.5 (t, *J* = 22.1 Hz, CF_2CH_3); **HRMS** (ESI^+) *m/z* calc. for $C_{12}H_{11}OF_2S$ $[M+H]^+$ 241.0420, found 241.0491.

5. Enantiomeric excess analysis

5.1 1-((1,1-Difluoroethyl)sulfinyl)-4-methoxybenzene (6)

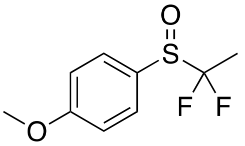

HPLC data for compound **6**: Chiralcel ID (95:5 hexane:IPA, flow rate 1 mL/min, 254 nm, 30 °C), t_R (A): 15.6 min, t_R (B): 16.5 min; 20:80 ee.

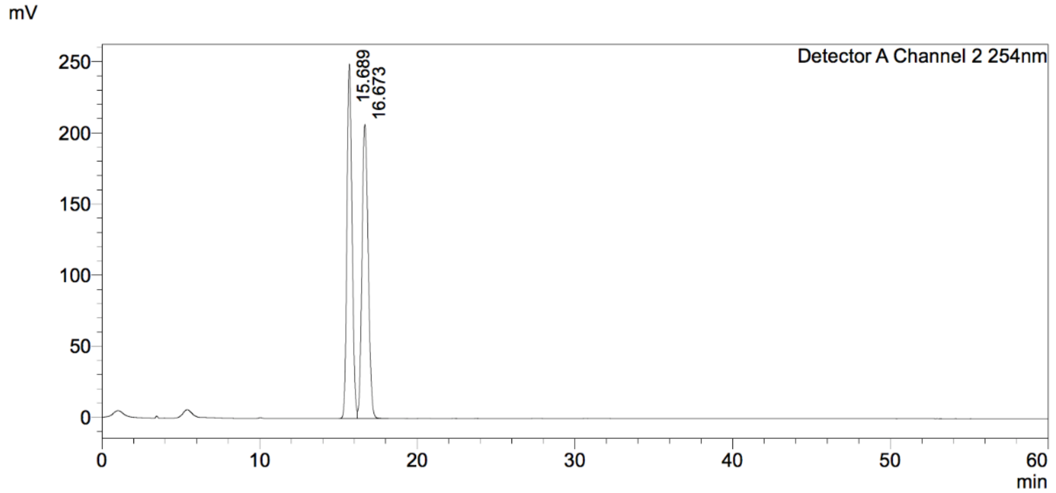

<Peak Table>

Detector A Channel 2 254nm

Peak#	Ret. Time	Area%
1	15.689	49.906
2	16.673	50.094
Total		100.000

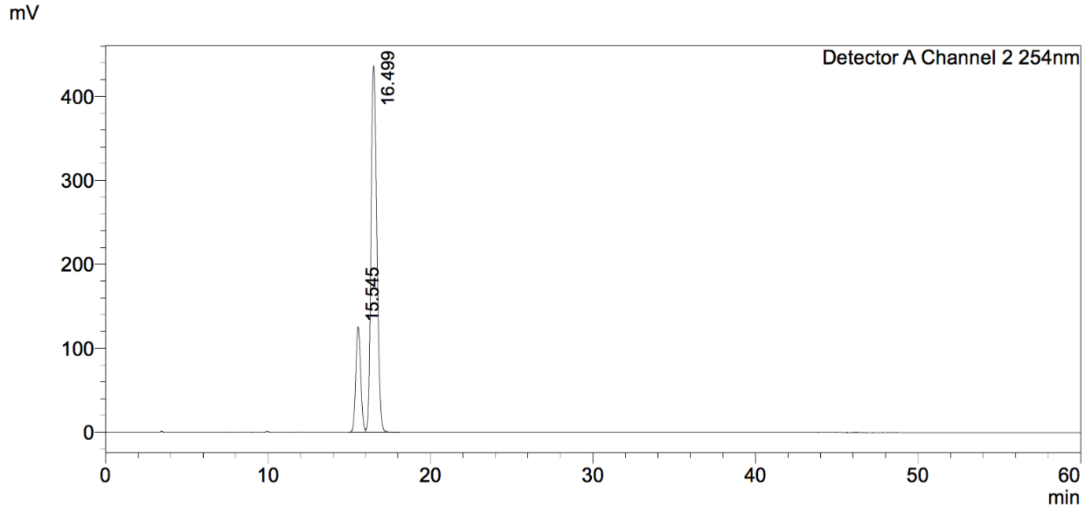

<Peak Table>

Detector A Channel 2 254nm

Peak#	Ret. Time	Area%
1	15.545	19.640
2	16.499	80.360
Total		100.000

5.2 2-((1,1-Difluoroethyl)sulfinyl)naphthalene (11)

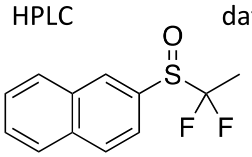

HPLC data for compound **11**: Chiralcel IC (95:5 hexane:IPA, flow rate 1 mL/min, 254 nm, 30 °C), t_R (A): 18.9 min, t_R (B): 16.5 min; 23:77 ee.

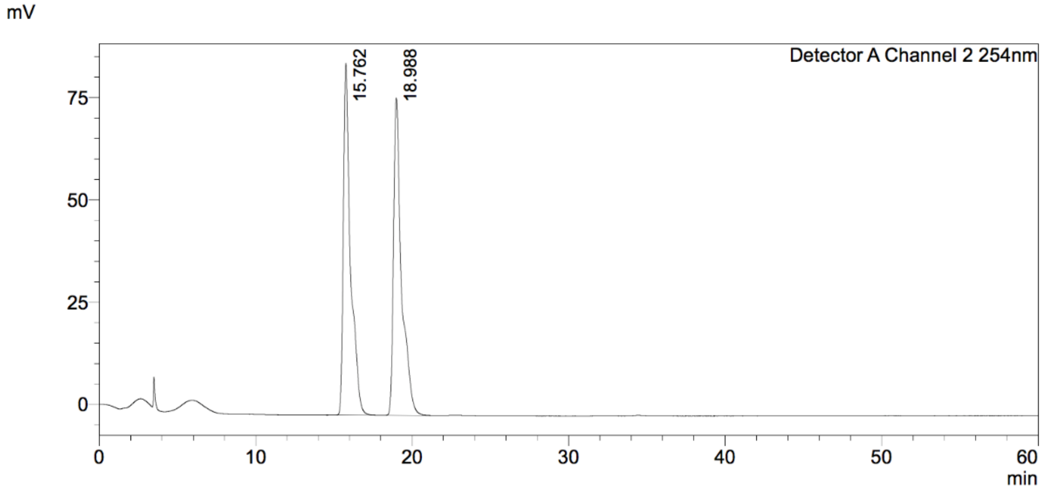

<Peak Table>

Detector A Channel 2 254nm

Peak#	Ret. Time	Area%
1	15.762	49.991
2	18.988	50.009
Total		100.000

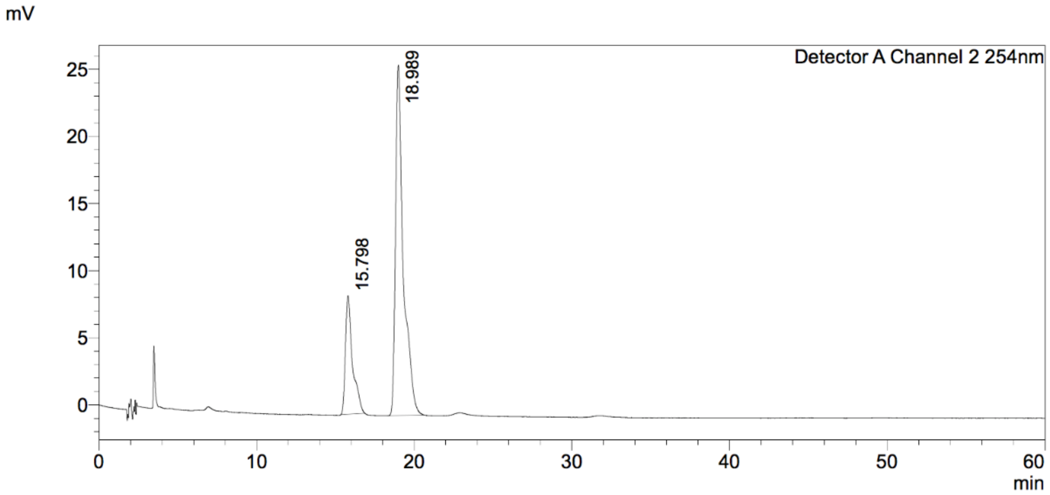

<Peak Table>

Detector A Channel 2 254nm

Peak#	Ret. Time	Area%
1	15.798	23.390
2	18.989	76.610
Total		100.000

6. Single crystal X-ray analysis

4-((1,1-Difluoroethyl)sulfinyl)phenol (7)

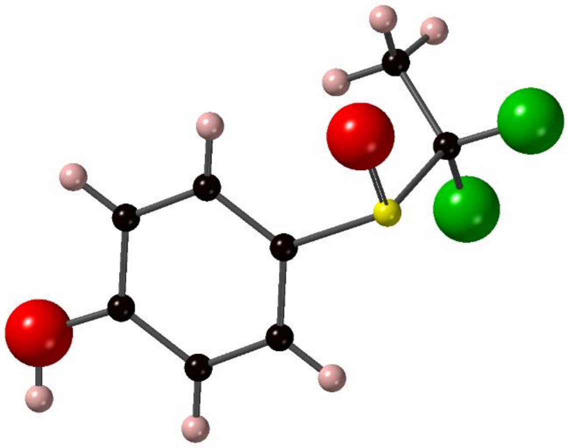

Code: agdh14

Structure Type: Crystal

Chemical Formula: $C_8 H_8 F_2 O_2 S$

Display Formula: $C_8 H_8 F_2 O_2 S$

Spacegroup: $P\ 2_1\ 2_1\ 2_1$

(Allows Chirality)

Crystal System: Orthorhombic

a: 8.5526 Å

b: 9.2693 Å

c: 10.9710 Å

Asymmetric Unit: 21 sites

Unit Cell: 84 sites per unit cell

Site Density: 0.0966 sites/Å^3

Visible Atoms: 21

Cell Volume: 869.744 Å^3

Density: 1.5748 g/cm^3

7. NMR Spectra

1-((1,1-Difluoroethyl)sulfinyl)-4-methoxybenzene (6)

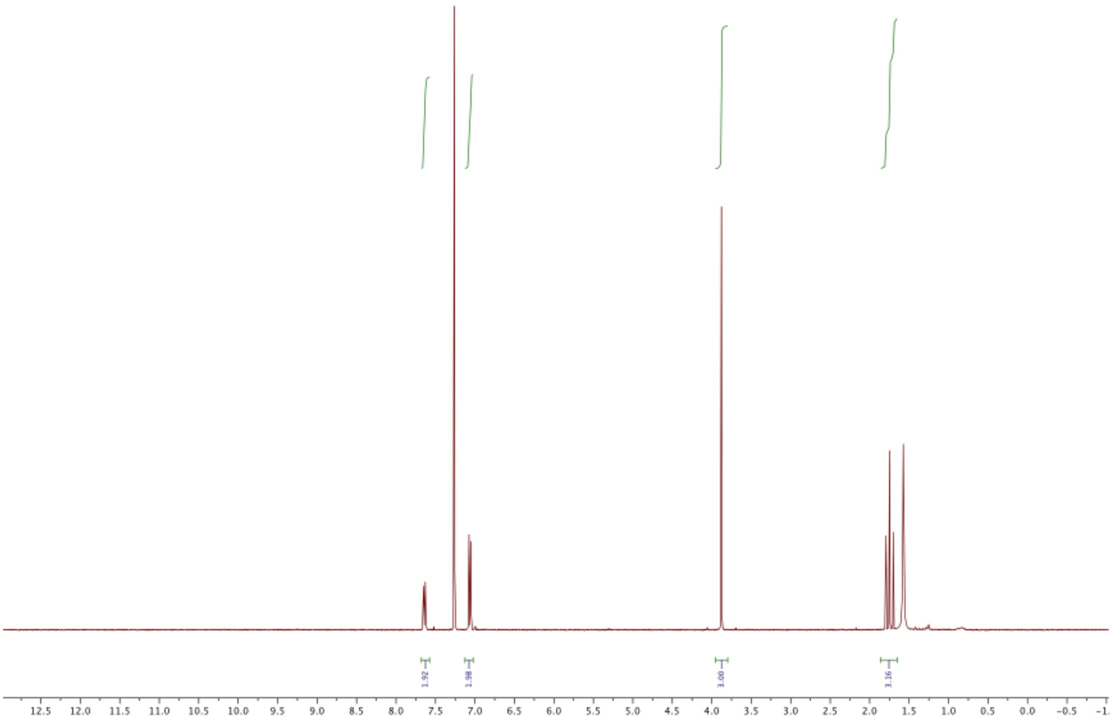

^{1}H-NMR

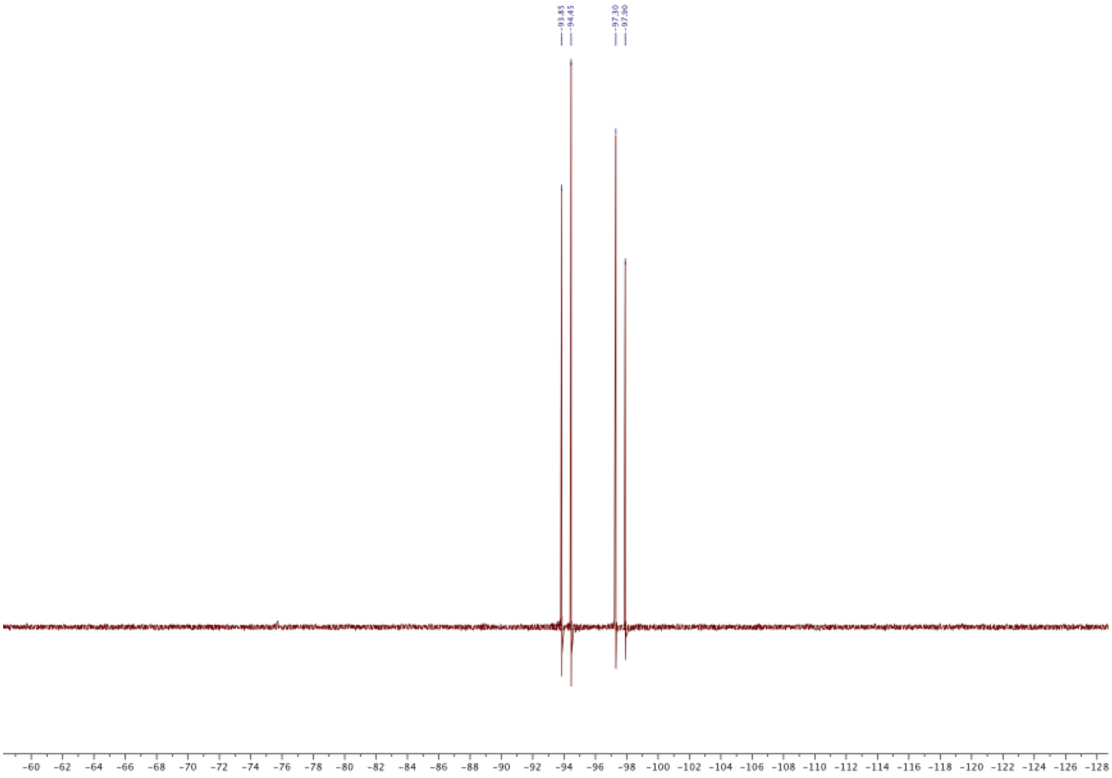

^{19}F-NMR

4-((1,1-Difluoroethyl)sulfinyl)phenol (7)

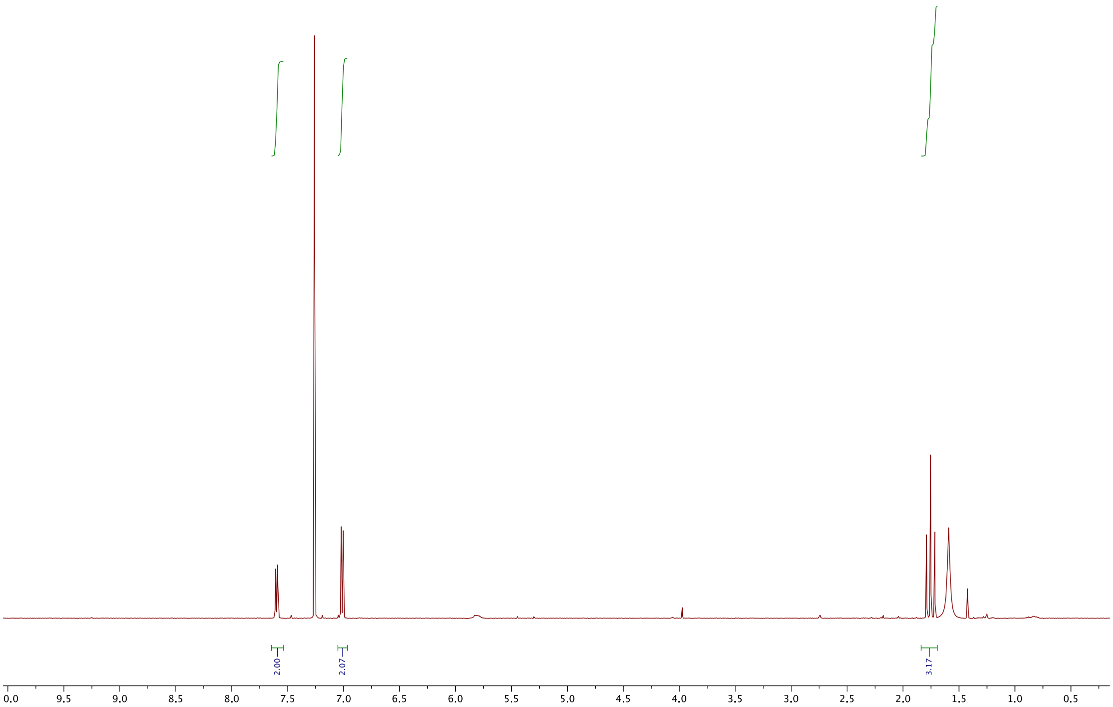

^{1}H NMR

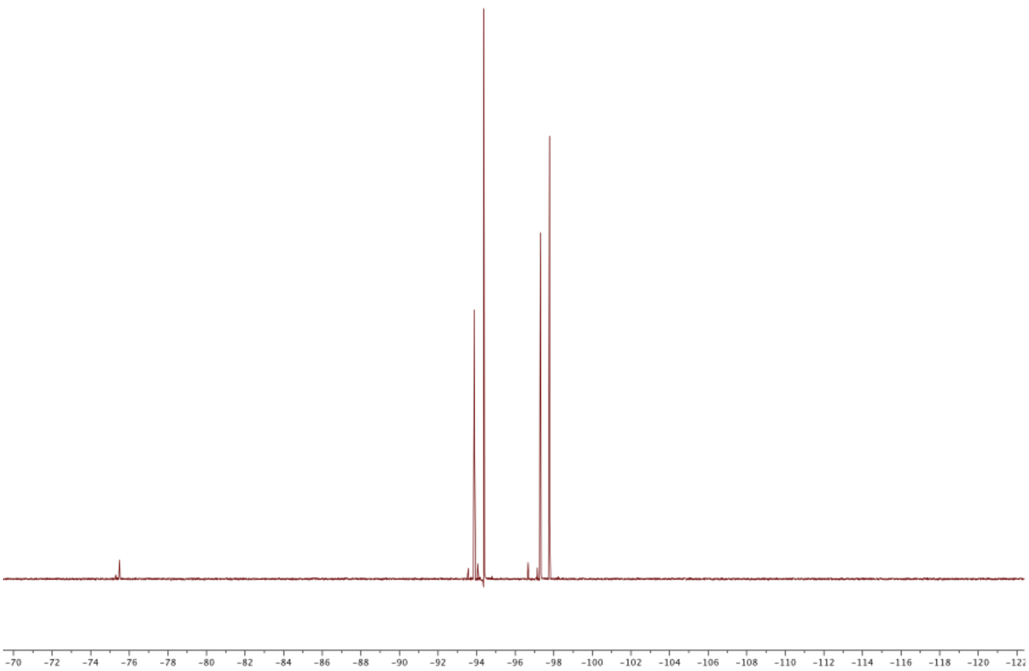

^{19}F NMR

4-((1,1-Difluoroethyl)sulfonyl)phenol (8)

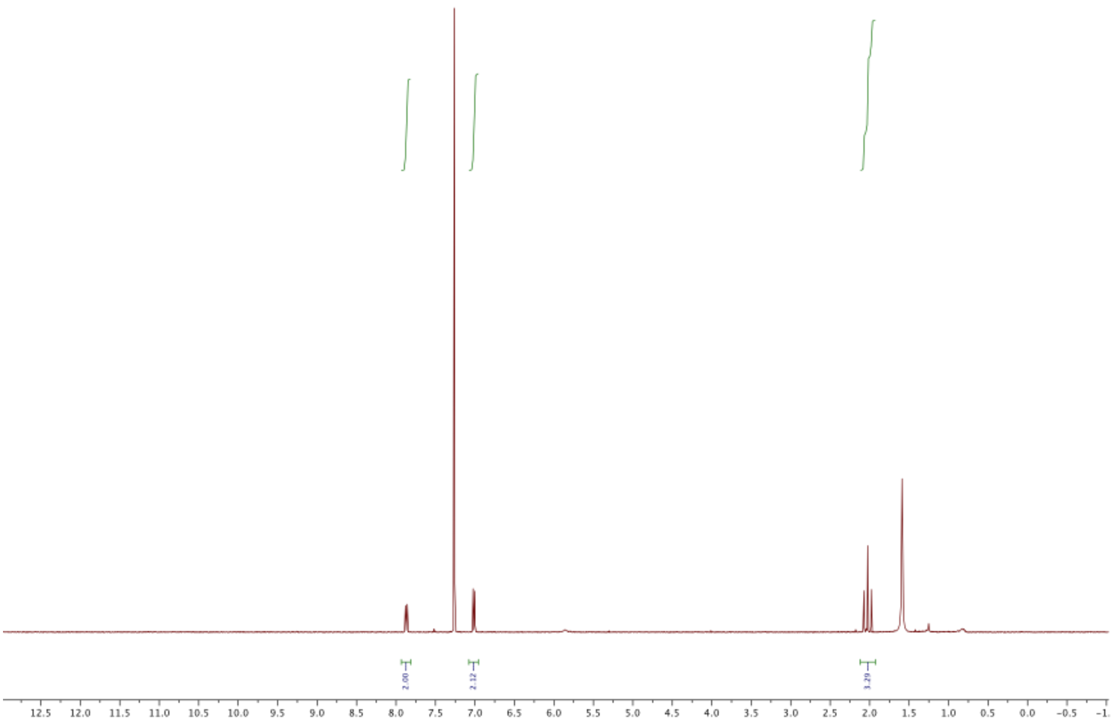

^{1}H NMR

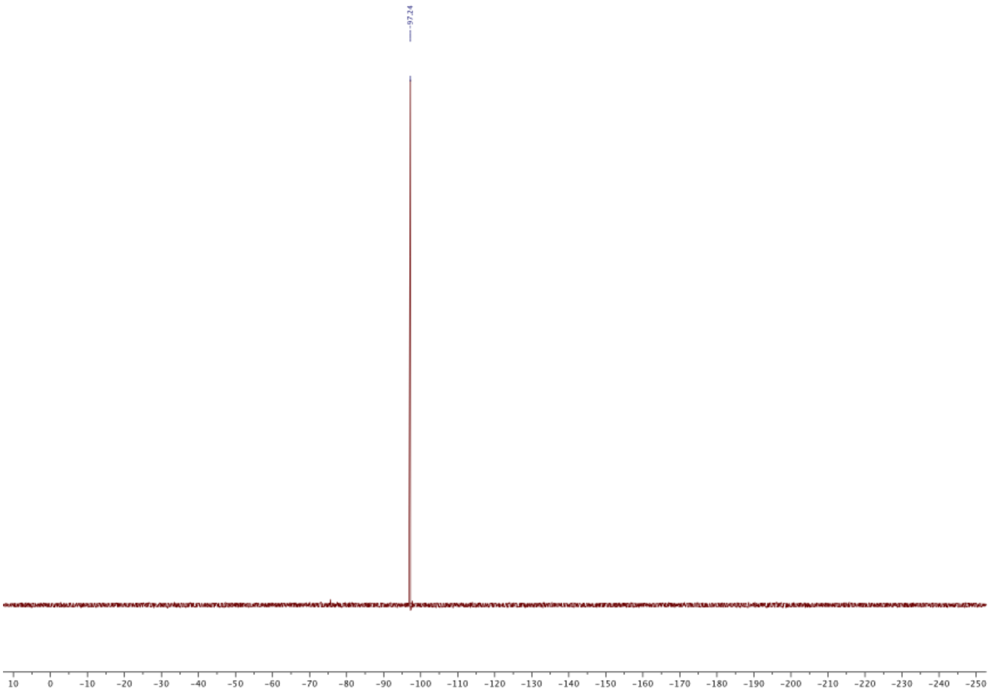

^{19}F NMR

1-((1,1-Difluoroethyl)sulfonyl)-4-methoxybenzene (9)

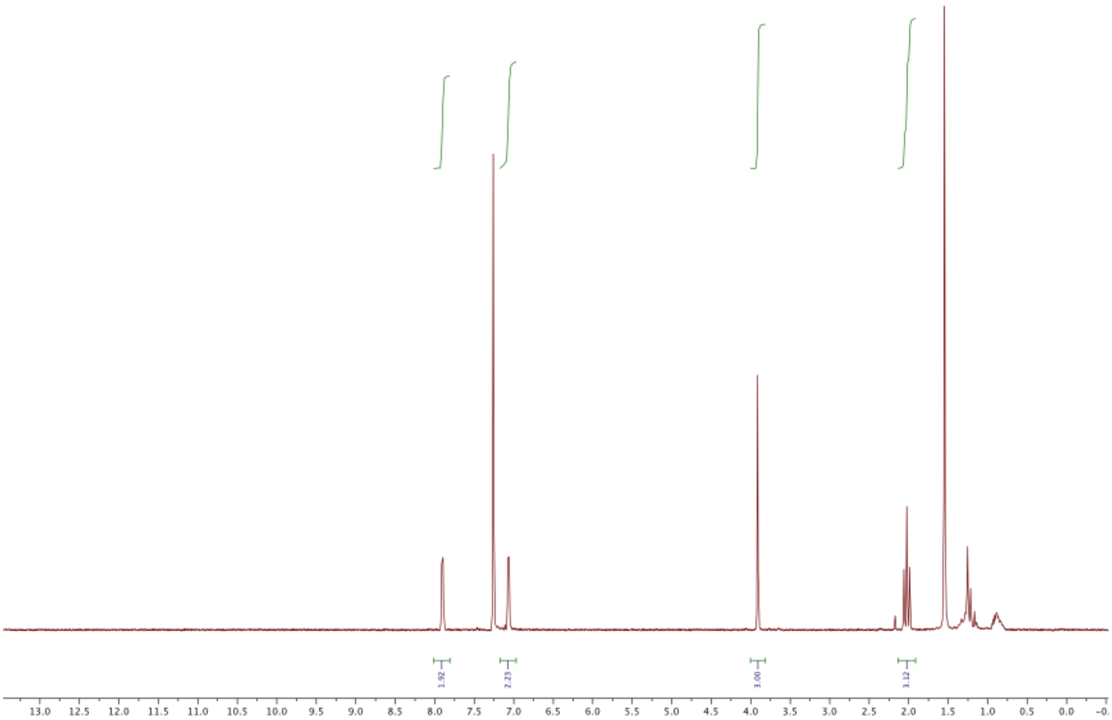

^{1}H NMR

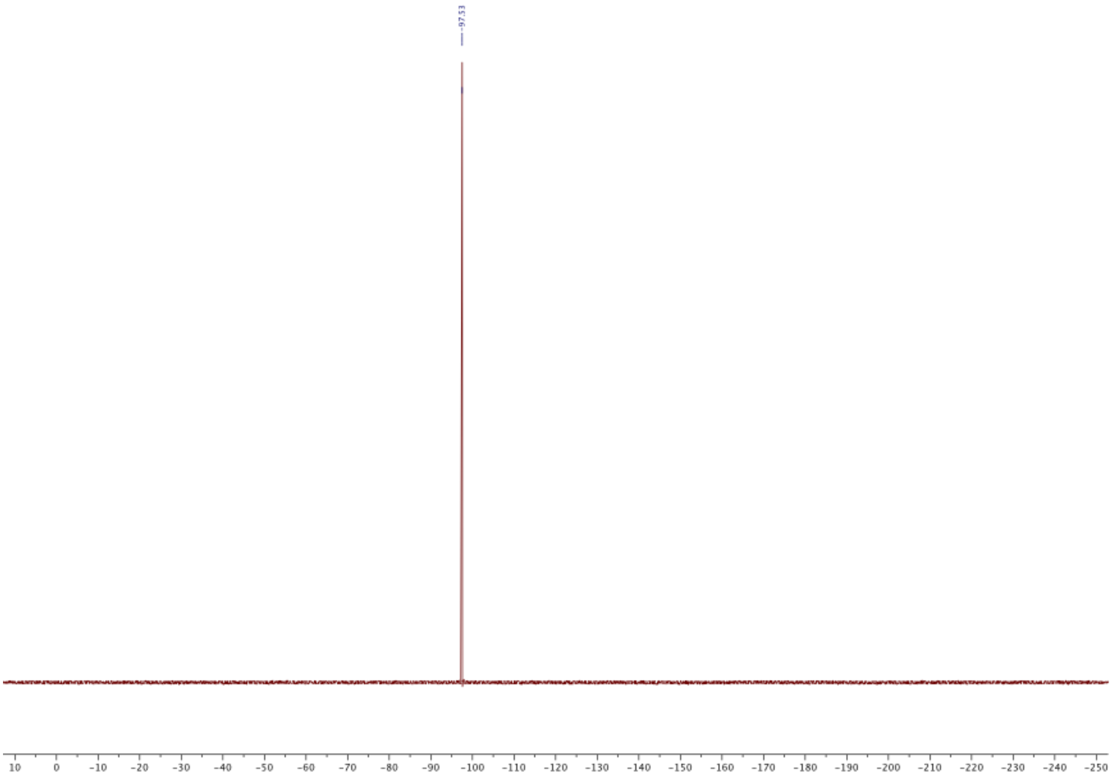

^{19}F NMR

2-((1,1-difluoroethyl)sulfinyl)naphthalene (11)

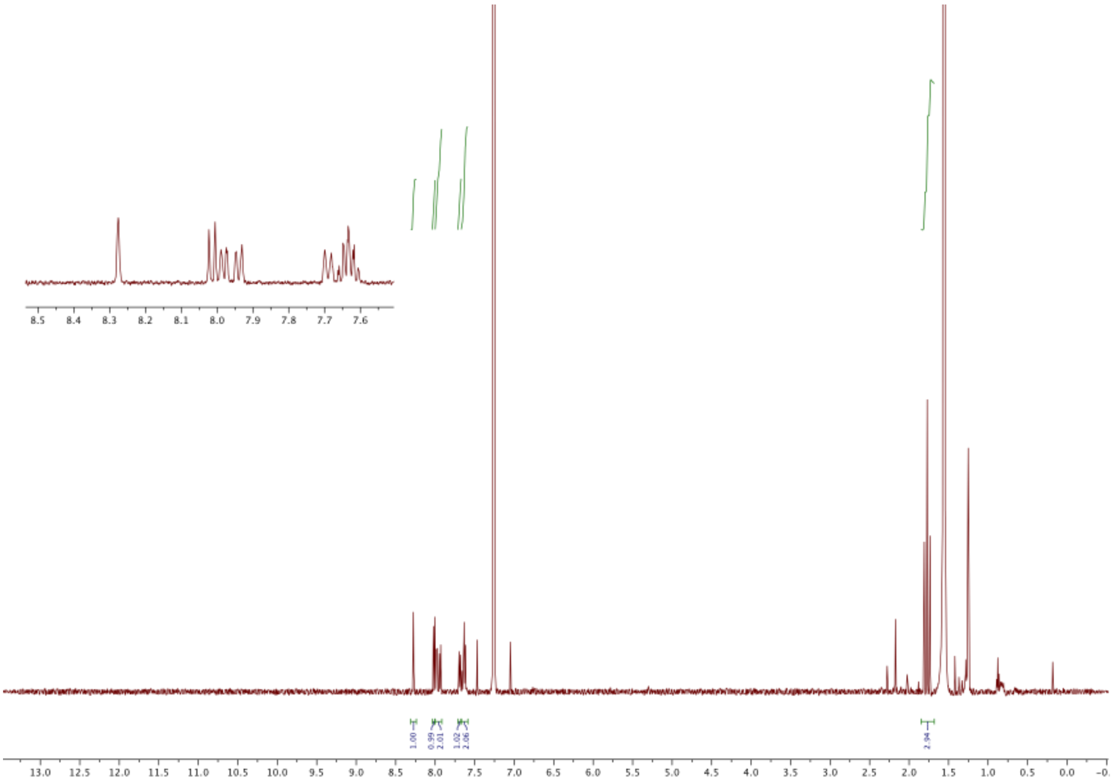

^{1}H NMR

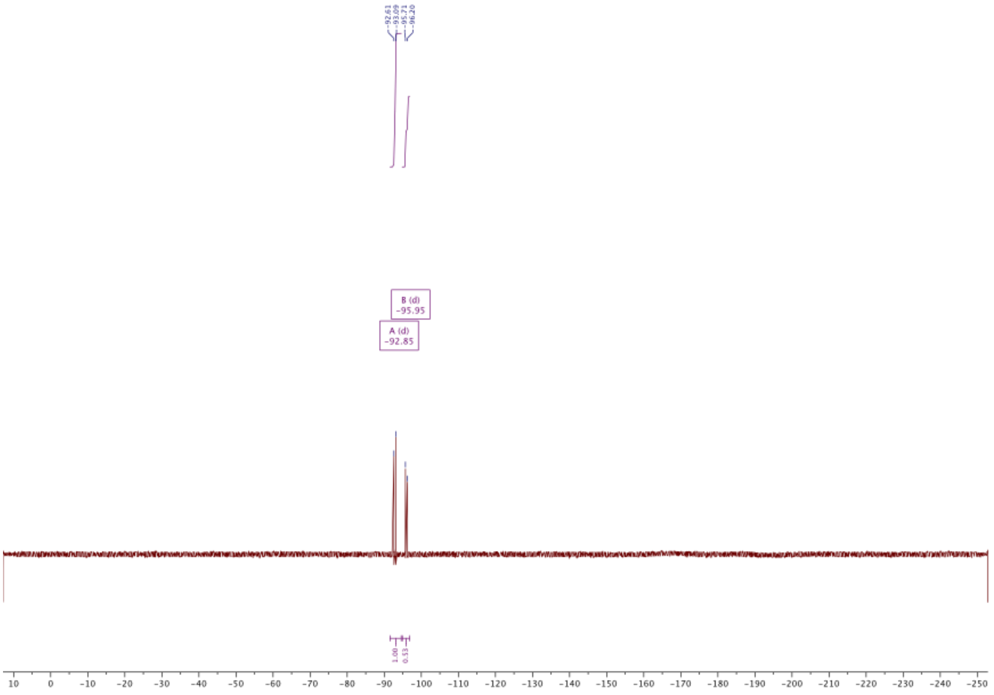

^{19}F NMR

6-((1,1-Difluoroethyl)sulfinyl)naphthalene-1,4-diol (12)

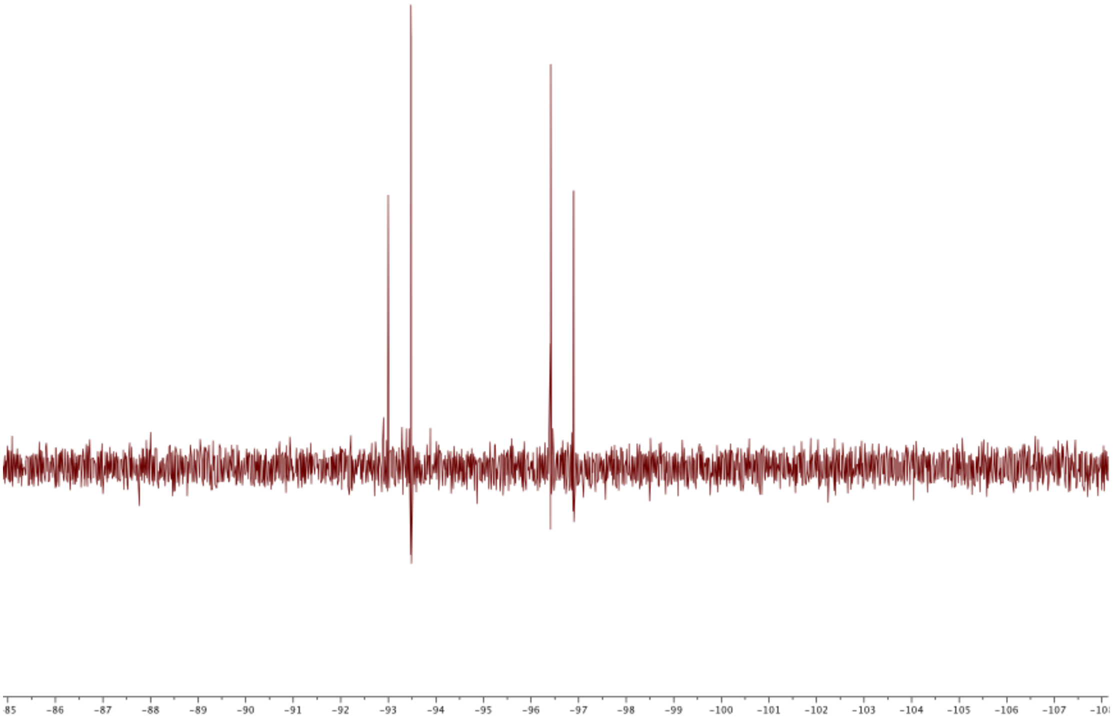

^{19}F NMR

(1,1-difluoroethyl)sulfinyl)naphthalene (13)

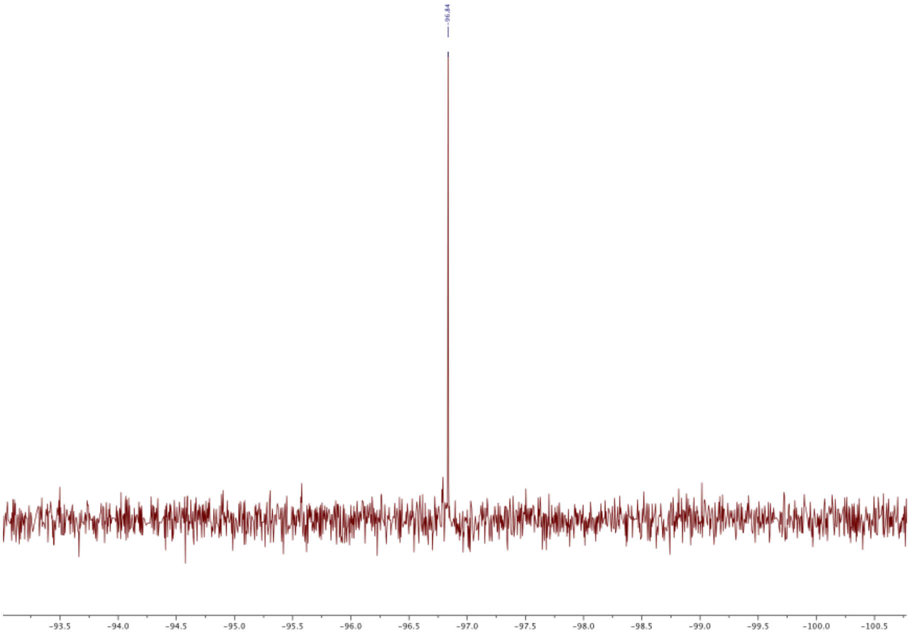

^{19}F NMR

4-Acetoxyphenol (15)

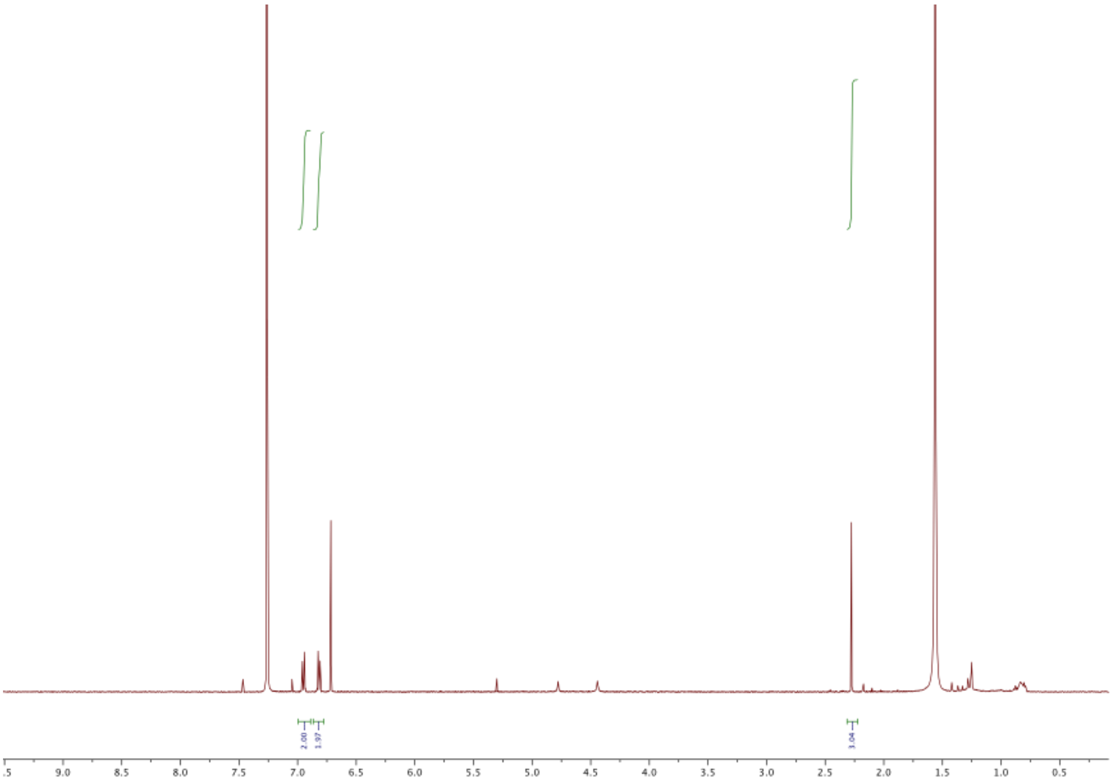

^{1}H NMR

4-(1,1-Difluoro)ethoxy phenol (16)

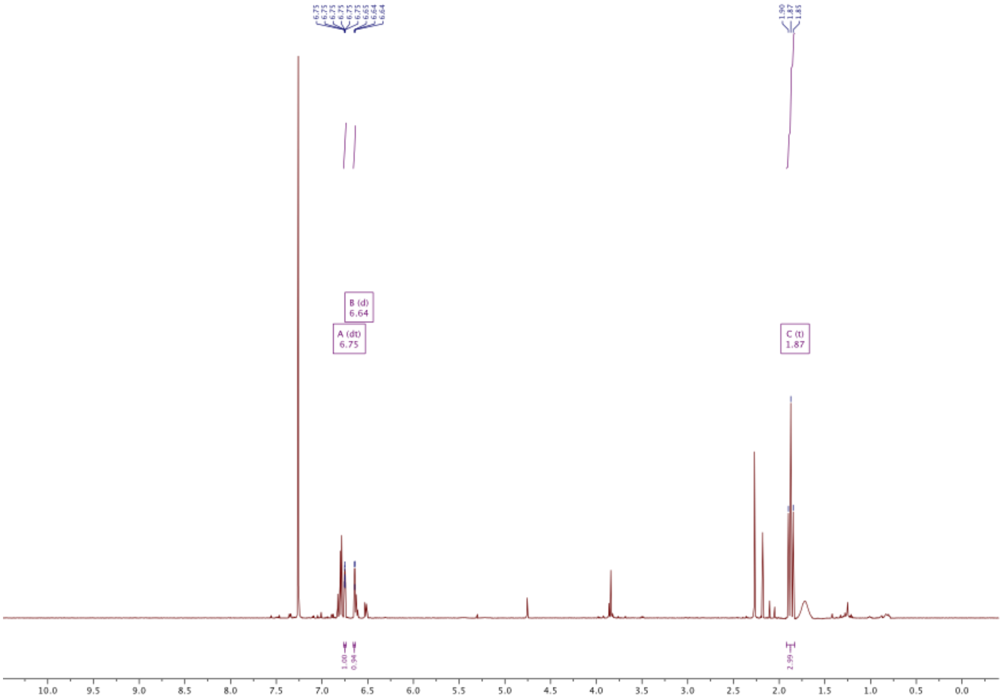

^{1}H NMR

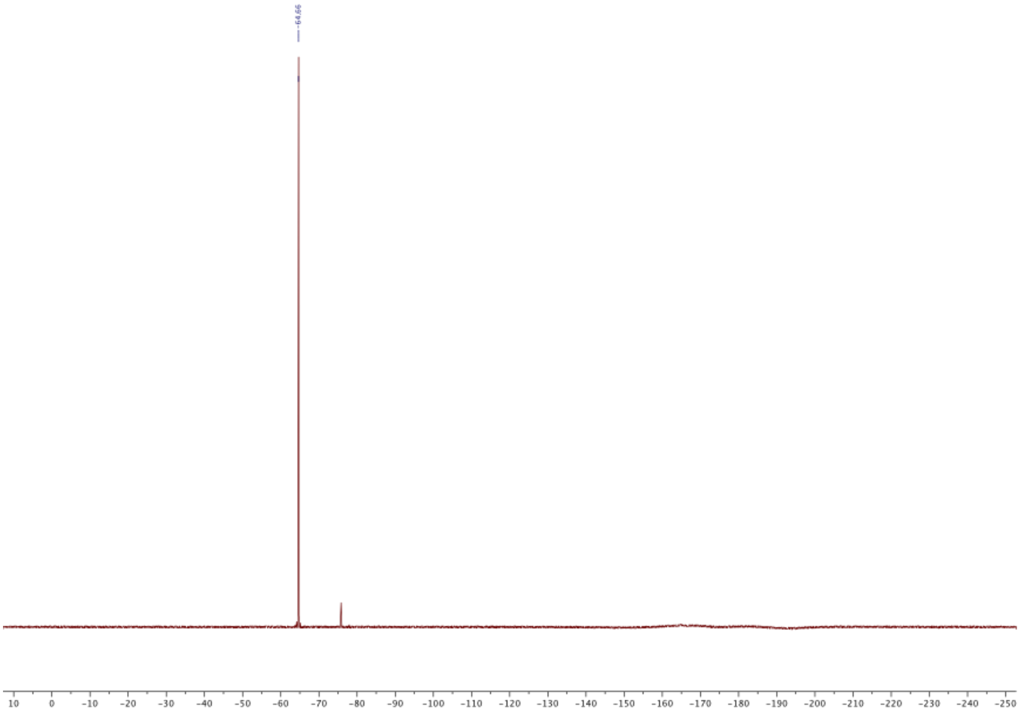

^{19}F NMR

8. References

Coombes CL, Moody CJ (2008) First syntheses of 2,2-dimethyl-7-(2′-methylbut-3′-en-2′-yl)-2*H*-chromen-6-ol and 2-(3′-Methylbut-2′-enyl)-5-(2′-methylbut-3′-en-2′-yl)-1,4-benzoquinone, novel prenylated quinone derivatives from the New Zealand brown alga *Perithalia capillaris*. J Org Chem 73:6758–6762. https://doi.org/10.1021/jo801057x

Tomita R, Al-Maharik N, Rodil A, Bühl M, O'Hagan D (2018) Synthesis of aryl α,α-difluoroethyl thioethers a novel structure motif in organic chemistry, and extending to aryl α,α-difluoro oxyethers. Org Biomol Chem 16:1113–1117. https://doi.org/10.1039/C7OB02987J
